# Butyrylcholinesterase genotype and enzyme activity in relation to Gulf War illness: preliminary evidence of gene-exposure interaction from a case–control study of 1991 Gulf War veterans

**DOI:** 10.1186/1476-069X-14-4

**Published:** 2015-01-09

**Authors:** Lea Steele, Oksana Lockridge, Mary M Gerkovich, Mary R Cook, Antonio Sastre

**Affiliations:** Veterans Health Research Program, Baylor University Institute of Biomedical Studies, One Bear Place # 97261, Waco, TX 76798 USA; The Eppley Institute, University of Nebraska Medical Center, Omaha, NE 68198 USA; Department of Biomedical and Health Informatics, University of Missouri-Kansas City School of Medicine, Kansas City, MO 64108 USA; MRIGlobal, Kansas City, MO 64110 USA; 3049D Trevor House Drive, Oakton, VA 22124 USA

**Keywords:** Butyrylcholinesterase, Gulf War illness, Gulf War veterans, Pyridostigmine bromide, Gene-environment interaction

## Abstract

**Background:**

Epidemiologic studies have implicated wartime exposures to acetylcholinesterase (AChE)-inhibiting chemicals as etiologic factors in Gulf War illness (GWI), the multisymptom condition linked to military service in the 1991 Gulf War. It is unclear, however, why some veterans developed GWI while others with similar exposures did not. Genetic variants of the enzyme butyrylcholinesterase (BChE) differ in their capacity for metabolizing AChE-inhibiting chemicals, and may confer differences in biological responses to these compounds. The current study assessed BChE enzyme activity and BChE genotype in 1991 Gulf War veterans to evaluate possible association of this enzyme with GWI.

**Methods:**

This case–control study evaluated a population-based sample of 304 Gulf War veterans (144 GWI cases, meeting Kansas GWI criteria, and 160 controls). BChE enzyme activity levels and genotype were compared, overall, in GWI cases and controls. Potential differences in risk associated with cholinergic-related exposures in theater were explored using stratified analyses to compare associations between GWI and exposures in BChE genetic and enzyme activity subgroups.

**Results:**

Overall, GWI cases and controls did not differ by mean BChE enzyme activity level or by BChE genotype. However, for the subgroup of Gulf War veterans with less common, generally less active, BChE genotypes (K/K, U/AK, U/A, A/F, AK/F), the association of wartime use of pyridostigmine bromide (PB) with GWI (OR = 40.00, p = 0.0005) was significantly greater than for veterans with the more common U/U and U/K genotypes (OR = 2.68, p = 0.0001).

**Conclusions:**

Study results provide preliminary evidence that military personnel with certain BChE genotypes who used PB during the 1991 Gulf War may have been at particularly high risk for developing GWI. Genetic differences in response to wartime exposures are potentially important factors in GWI etiology and should be further evaluated in conjunction with exposure effects.

## Background

Studies of veteran populations in the U.S. and other countries have consistently described a profile of chronic symptoms that afflict veterans of the 1991 Gulf War at significantly excess rates, compared to other veteran groups. This condition, commonly known as Gulf War illness (GWI), is estimated to affect between one fourth and one third of the nearly 700,000 U.S. veterans who served in the 1991 conflict [1–4]. Gulf War illness is characterized by multiple concurrent symptoms that include persistent headaches, cognitive difficulties, widespread pain, fatigue, gastrointestinal problems, and other chronic abnormalities—symptoms not accounted for by medical or psychiatric conditions routinely diagnosed in clinical practice. Longitudinal studies indicate that few veterans have recovered or substantially improved over time [2, 5, 6].

Despite extensive research related to the health of Gulf War veterans, a detailed understanding of the specific nature and causes of GWI has not yet been conclusively established. Diverse biological alterations have been described in veterans with Gulf War illness compared to healthy controls, including significant differences in brain structure and function [7–10] as well as autonomic [11–13], immune [14–17], and endocrine [18, 19] parameters. Epidemiologic studies of Gulf War veterans consistently indicate that GWI is not associated with combat stressors, when effects of other wartime exposures are taken into account, and that rates of psychiatric illness associated with the brief 1991 conflict are substantially lower than for other wars [2, 20, 21].

A variety of potentially hazardous chemicals encountered by some Gulf War military personnel have been suggested as causes or contributors to GWI. These include multiple exposures with the potential to cause adverse neurological effects, including low-level exposure to chemical nerve agents, use of pyridostigmine bromide (PB) pills given prophylactically as a protective measure against nerve agents, and extensive use of pesticides. [2, 21–23].

Few measurements were made during the war that documented the levels and concentrations of exposures experienced by individual veterans. However, useful information on deployment-related exposures has been provided by government investigations utilizing a variety of methods, including extensive command and field interviews, record reviews, event and exposure simulations, and modeling efforts [2]. Estimates provided by the Department of Defense (DOD) indicate that about 100,000 U.S. military personnel were potentially exposed, in March 1991, to low levels of the nerve agents sarin and cyclosarin in connection with demolitions at a large Iraqi munitions compound near Khamisiyah, Iraq [24]. Government reports also indicate that about half of American ground troops took PB tablets in varying amounts, and that military personnel used a wide range of pesticides and insect repellants in different combinations, often at excessive levels [22, 23].

Sarin and cyclosarin are organophosphate compounds, PB is a carbamate compound, and the many pesticides used by military personnel in the Gulf War included a variety of both organophosphates and carbamates. These chemicals are acetylcholinesterase (AChE) inhibitors; that is, their chemical action interferes with breakdown of the neurotransmitter acetylcholine. These compounds can be acutely toxic, even fatal, at sufficiently high doses. But their long-term effects, particularly in relation to repeated, lower-dose exposures, are less well understood. Epidemiologic studies of Gulf War veterans consistently indicate that veterans who report using PB and pesticides have significantly higher rates of GWI than veterans who did not use these compounds in theater [21, 25–27].

An important question concerning GWI relates to why some personnel developed chronic symptoms after the war while others with similar wartime experiences and exposures remained healthy. One possibility is that vulnerability to certain deployment-related exposures differed as a result of individual variability in the biological processes that neutralize these exposures and confer protection from acute and/or chronic adverse effects. Recent reports from both the federal Research Advisory Committee on Gulf War Veterans’ Illnesses [20] and the Institute of Medicine [4] have suggested that genetic factors may have played a role in the development of GWI and have urged additional research to evaluate this possibility.

Acetylcholinesterase-inhibiting chemical exposures interact with a variety of circulating enzymes in humans, including two cholinesterases—butyrylcholinesterase (BChE) and AChE. Butyrylcholinesterase is present at more than 10 times the level of AChE in whole blood, but its physiological functions are not completely understood. It is known to provide protection from adverse effects of carbamates, organophosphates, and other chemicals by acting as a scavenger, binding them molecule-for-molecule, thereby sparing circulating levels of AChE [28].

There is one gene for BChE, located on chromosome 3q26, with multiple nucleotide variations identified at this locus [28]. The wild type “U” allele is most common, followed by the K, A, and F mutations. The less common variants are associated, on average, with lower enzyme activity levels and/or binding affinity for AChE-inhibiting compounds, rendering individuals especially sensitive to a number of chemicals [28–30]. Individuals who are AA homozygotes, for example, experience prolonged apnea and paralysis—up to two hours—if given the muscle relaxant succinylcholine for surgery at doses that normally act only for several minutes [30]. Limited evidence also suggests that some BChE genetic variants may be more vulnerable to adverse effects of AChE inhibitors, including PB [28, 31].

We report here on results from a case–control study that assessed BChE genotype and enzyme activity in veterans of the 1991 Gulf War. Data for the study were collected in 2001 in connection with a multipart project that evaluated deployment experiences, genetic variability, and autonomic nervous system function in veterans of the 1991 Gulf War.

## Methods

### Study design and participants

The study utilized a case–control design to evaluate BChE activity and genotype in relation to GWI and deployment characteristics in a population-based sample of 304 Gulf War veterans. Detailed information on the study sample and data collection have previously been described [21]. Study participants included 144 GWI cases and 160 controls, with cases defined according to Kansas GWI criteria [1]. Briefly, the Kansas GWI case definition requires that veterans endorse moderately severe or multiple chronic symptoms in at least three of six defined symptom domains. Veterans are excluded as GWI cases if they report being diagnosed with any from a list of chronic medical conditions that might account for their symptoms, or severe psychiatric conditions that might preclude their ability to accurately report symptoms. Veteran controls had insufficient symptoms to meet GWI case criteria, and reported no exclusionary medical or psychiatric diagnoses. The study questionnaire also included symptom questions that allowed identification of veterans who met case criteria for “chronic multisymptom illness (CMI),” as defined by Fukuda et al. [32] at the U.S. Centers for Disease Control and Prevention, sometimes referred to as the “CDC” case definition.

In addition to symptoms and medical conditions, veterans provided information on their demographic and military characteristics, on 19 experiences and exposures potentially encountered during their 1990–1991 deployment, and on the general locations in which they served in the Gulf War theater of operations.

In conducting this research, investigators complied with all applicable U.S. regulations regarding the protection of human subjects. The study protocol was approved by Institutional Review Boards of the Kansas Department of Health and Environment and Midwest Research Institute, and by the Army’s Office of Human Research Protections. Veteran participants gave both oral and written informed consent prior to enrolling in the study.

### Laboratory methods

Two 9.5 mL tubes of blood were collected from each veteran to determine BChE enzyme activity level and genotype. Phenotype was determined by measuring serum enzyme activity with benzoylcholine in Na/K phosphate buffer, as previously described [33]. Inhibition of activity by dibucaine was used to identify “atypical” (A) and fluoride-resistant (F) phenotypes. Where unusual dibucaine inhibition levels were observed, inhibition by 50 μM sodium fluoride was measured to distinguish between UA, UF, AF, FF, and FS phenotypes [34, 35].

DNA was isolated and amplified by polymerase chain reaction, followed by restriction enzyme digestion, to determine genotype at the BChE polymorphic site Ala/Thr 539 (K variant). Amplification created a Mae III restriction site when the K-variant ACA codon (Thr 539) was present [35]. DNA from samples that phenotyped as heterozygous for the BChE F variant were amplified and sequenced to determine which of three reported DNA mutations were responsible for the fluoride resistance [36, 37]. Similarly, DNA from samples that phenotyped as heterozygous for the atypical variant (Asp70Gly) were amplified and sequenced to confirm the presence of the codon for Gly70 [38]. Laboratory analyses were performed by technicians who had no information on characteristics of veterans’ health or military service.

### Data analyses

Mean BChE enzyme activity levels were determined for each genetic type identified in the sample, for GWI cases and controls, and for demographic subgroups. Mean values were compared using Student’s t-tests. Proportional comparisons between veteran subgroups were assessed using chi-square statistics except as noted.

Associations between GWI and each “cholinergic” exposure queried for the study (that is, exposures with the potential for cholinergic effects) were evaluated in veteran subgroups defined by BChE activity and genotype. Comparably-sized BChE activity subgroups were defined according to whether veterans’ BChE activity level, measured at the time of the study, was equal to/above vs. below the median level for the sample as a whole (1.09 μmoles benzoylcholine per minute per mL serum). Prevalence odds ratios (ORs) for GWI/exposure associations were calculated separately for BChE activity subgroups and compared across strata.

Similar analyses compared associations between GWI and exposures in BChE genetic subgroups. Because of the relatively small number of individuals with each of the less common, and generally less active, BChE genotypes (K/K, U/AK, U/A, A/F, AK/F) in the sample, veterans with these genotypes were combined into a single BChE “less common variants” (BChE-LCV) subgroup, for comparison to veterans with U/U and U/K genotypes. Prevalence odds ratios (ORs) for GWI/exposure associations were calculated separately for BChE genetic subgroups and compared across strata.

To assess possible confounding issues that might underlie the gene-exposure interaction identified by the study, post-hoc analyses were conducted to determine whether characteristics and exposures reported by the BChE-LCV genetic subgroup differed from those of other veterans in the study. Additional post-hoc analyses assessed whether the identified interaction might have been an anomaly of the Kansas GWI case criteria used for the study. For this assessment, symptom data were used to “reassign” case/control status for all 304 veteran subjects, with case status determined using the CMI criteria of Fukuda et al. [32]. Prevalence odds ratios (ORs) were again calculated separately for BChE genetic subgroups to assess the association of illness with exposure in CMI cases and controls. All statistical analyses were conducted using SAS statistical software, version 9.2 [39].

## Results

### Study population

Health, demographic, and military characteristics of the study population are described in detail elsewhere [21]. Gulf War illness cases were similar to controls with respect to sex (cases: 8% female; controls: 7% female; p = 0.80) and age (mean age cases: 37.4; mean age controls: 36.9; p = 0.53) at study intake, but differed significantly by race (cases: 83% white; controls 94% white; p = 0.01).

### BChE activity and genotype in Gulf War veterans

Mean BChE enzyme activity levels are shown in Table 1 for all veterans combined, for each BChE genotype in the sample, for demographic subgroups, and for GWI cases and controls. As expected, the BChE-LCV subgroup of veterans exhibited, on average, significantly lower BChE activity levels than veterans with U/U and U/K genotypes. Enzyme activity did not vary significantly by race or by age. Similar to the general population [40], female Gulf War veterans had significantly lower BChE activity than males. The mean BChE activity level of GWI cases was nearly identical to that of controls.

**Table 1:** **Butyrylcholinesterase (BChE) enzyme activity in Gulf War veterans**

	n	BChE mean (SD) activity ^*^
**All veterans in study**	304	1.10 (0.26)
**BChE genotype**		
U/U	189	1.19 (0.24)
U/K	87	1.01 (0.21)
K/K	13	0.80 (0.15)
U/AK	10	0.76 (0.18)
U/A	3	1.03 (0.12)
A/F	1	0.92
AK/F	1	0.69
**Common variants combined** (U/U and U/K)	276	1.13 (0.24)
**Less common variants combined** (K/K, U/AK, U/A, A/F, AK/F)	28	0.81 (0.17)^↑^
**Race**		
White	265	1.10 (0.26)
Black	26	1.07 (0.24)
Other	8	1.12 (0.23)
**Sex**		
Male	282	1.11 (0.25)
Female	22	0.95 (0.26)^↑↑^
**Age at time of study**		
29-39	189	1.10 (0.25)
40-49	77	1.14 (0.27)
50+	38	1.05 (0.23)
**Gulf War illness case status**		
Gulf War illness cases	144	1.10 (0.24)
Gulf War veteran controls	160	1.10 (0.27)

BChE = butyrylcholinesterase, SD = standard deviation.

^*^Mean enzyme activity expressed in μmoles benzoylcholine hydrolyzed per minute per mL of serum.

^↑^Mean enzyme activity differs significantly from U/U and U/K (p < 0.001).

^↑↑^Mean enzyme activity differs significantly from males (p = 0.003).

When the sample was evaluated overall, the BChE genetic profiles of GWI cases and controls were also nearly identical (Table 2). That is, no significant association between GWI case status and BChE genotype was found, either in the distribution of specific BChE variants or in the overall proportion of cases and controls with less common BChE genotypes.

**Table 2:** **Distribution of butyrylcholinesterase genotype in Gulf War illness cases and controls**

	Total sample (n = 304)		GWI cases (n = 144)		Controls (n = 160)	
BChE genotype	n	(%)	n	(%)	n	(%)
U/U	189	(62%)	89	(62%)	100	(62%)
U/K	87	(29%)	41	(28%)	46	(29%)
K/K	13	(4%)	7	(5%)	6	(4%)
U/AK	10	(3%)	5	(3%)	5	(3%)
U/A	3	(1%)	1	(<1%)	2	(1%)
A/F	1	(<1%)	0	(0%)	1	(<1%)
AK/F	1	(<1%)	1	(<1%)	0	(0%)
Less common variants combined (K/K, U/AK, U/A. A/F, AK/F)	28	(9%)	14	(10%)	14	(9%)

BChE = butyrylcholinesterase, GWI = Gulf War illness.

### Association between GWI and exposures in relation to BChE activity and genotype

We assessed whether BChE variability played a role in observed associations between GWI and deployment exposures that potentially had cholinergic effects. This included all variables related to pesticide exposures and PB use, and one variable possibly related to low-level nerve agent exposure (hearing chemical alarms). No significant differences were identified between Gulf War veteran subgroups defined by BChE enzyme activity. That is, associations between GWI and exposures were similar in veterans with lower versus higher BChE enzyme activity measured at the time of the study (results not shown in tables).

In evaluating associations between GWI and cholinergic exposures in relation to BChE genotype, there were no significant differences between U/U and U/K genotype subgroups (Table 3). For the BChE-LCV subgroup, however, GWI risk was dramatically elevated for veterans who reported taking PB pills during deployment (OR = 40.00, p = 0.0005, Fisher’s exact test). Use of PB pills was also associated with GWI among veterans with U/U and U/K genotypes, but to a much lesser degree (OR = 2.68 for U/U and U/K subgroups combined, p = 0.0001). The difference between ORs for veterans in the BChE-LCV subgroup and veterans with U/U and U/K genotypes was statistically significant (p = 0.019, Breslow-Day test for homogeneity of ORs), indicating a significant interaction between exposure and BChE genotype. This interaction indicates a substantially increased risk for GWI for the subgroup of Gulf War veterans with less common BChE genotypes (K/K, U/AK, U/A, A/F, and AK/F) who used PB during deployment.

**Table 3:** **Association of Gulf War illness with exposures with potential cholinergic effects, by butyrylcholinesterase genetic subgroup**

	Butyrylcholinesterase genetic subgroup								
	U/U homozygotes (n = 89 GWI cases, 100 controls)			U/K heterozygotes (n = 41 GWI cases, 46 controls)			Less common BChE variants (K/K, U/AK, U/A, A/F, AK/F) (n = 14 GWI cases, 14 controls)		
Experience/exposure	% cases exposed	% controls exposed	OR ^*^ (95% C.I.)	% cases exposed	% controls exposed	OR ^*^ (95% C.I.)	% cases exposed	% controls exposed	OR ^*^ (95% C.I.)
Took PB (pyridostigmine bromide) pills	67%	48%	2.25 (1.23–4.11)	74%	41%	3.98 (1.57–10.1)	92%	23%	**40.0** ^↑^ (3.58–447)
Wore uniforms treated with pesticides	22%	8%	3.07 (1.27–7.44)	37%	11%	4.92 (1.59–15.2)	29%	8%	4.80 (0.46–50.2)
Used pesticide cream or spray on skin	55%	31%	2.75 (1.50–5.02)	62%	28%	4.23 (1.71–10.5)	50%	43%	1.33 (0.31–5.91)
Living area sprayed with pesticides	21%	18%	1.23 (0.59–2.57)	24%	17%	1.47 (0.51–4.29)	21%	14%	1.64 (0.23–11.7)
Heard chemical alarms sounded	57%	57%	1.02 (0.57–1.82)	62%	44%	2.00 (0.84–4.79)	64%	50%	1.80 (0.40–8.18)

BChE = butyrylcholinesterase, GWI = Gulf War illness, OR = prevalence odds ratio, C.I. = confidence interval.

^*^OR compares GWI cases with controls within each genetic subgroup.

^↑^OR for veterans with less common BChE variant genotypes differs significantly from OR for veterans with U/U and U/K genotypes, p = 0.019.

No other significant interactions were identified in relation to BChE genotype. That is, the association of GWI with veteran-reported use of pesticides and experiences possibly linked to chemical weapons exposures did not differ among veterans in the U/U, U/K, and BChE-LCV genetic subgroups.

The striking interaction between BChE genotype and the use of PB pills during deployment prompted additional evaluations to assess whether the observed effect might have been the result of other factors, such as an inconsistency in the pattern of exposures reported by BChE variants, or an anomaly of the GWI case definition used for the study. Overall, veterans in the BChE-LCV subgroup were nearly identical to veterans in the other two subgroups with respect to sex, age, education, rank, branch of service, deployment locations, and proportion reporting deployment exposures. For example, 58% of veterans in the BChE-LCV subgroup reported using PB pills, compared to 57% of those with U/U and U/K genotypes (p = 0.93). Similarly, 19% of the BChE-LCV subgroup reported wearing uniforms treated with pesticides, compared to 18% of veterans with U/U and U/K genotypes (p = 0.90).

Additional analyses indicated that the elevated GWI risk associated with the use of PB by the BChE-LCV subgroup of veterans was not uniquely tied to the Kansas GWI case definition (Table 4). A similar pattern was observed when case status was reassigned, based on the chronic multisymptom illness criteria of Fukuda et al. [32], yielding 187 cases and 117 controls. Using either case definition, chronic symptomatic illness was significantly associated with use of PB pills for the sample as a whole. However, the PB-illness association was substantially more pronounced in the subgroup of veterans with less common, and generally slower acting BChE genotypes (K/K, U/AK, U/A, A/F, and AK/F).

**Table 4:** **Association of pyridostigmine bromide use with chronic illness in BChE subgroups: evaluation using two case definitions**

	Use of PB by all Gulf War veterans (n = 304)			Use of PB by butyrylcholinesterase genetic subgroups					
				Common BChE variants (U/U and U/K) (n = 276)			Less common BChE variants (K/K, U/AK, U/A, A/F, AK/F) (n = 28)		
Case definition	% cases exposed	% controls exposed	OR (95% C.I.)	% cases exposed	% controls exposed	OR (95% C.I.)	% cases exposed	% controls exposed	OR (95% C.I.)
GWI (Kansas case definition)^*^	72%	44%	3.21 (1.97–5.24)	69%	46%	2.68 (1.62–4.44)	92%	23%	40.00 (3.58–447)
CMI (CDC case definition)^↑^	63%	46%	1.99 (1.23–3.22)	62%	49%	1.73 (1.05–2.84)	78%	22%	11.37 (1.65–78.4)

BChE = butyrylcholinesterase, PB = pyridostigmine bromide, CDC = U.S. Centers for Disease Control and Prevention, OR = prevalence odds ratio, C.I. = confidence interval.

^*^Gulf War illness, as defined in Steele [1].

^↑^Chronic multisymptom illness, as defined in Fukuda et al. [32].

## Discussion

More than 23 years after the cease fire that ended Operation Desert Storm in 1991, our understanding of the specific factors that led to GWI remains incomplete. The present study provides the first direct evidence suggesting that interaction between genetic factors and a chemical exposure associated with Gulf War service may have contributed to the development of GWI. Specifically, the subgroup of Gulf War veterans in our study with less common, and generally less active, genetic variants of the enzyme BChE were at substantially increased risk for GWI if they took PB pills during deployment. This genetic subgroup constituted 9% of the veterans in our sample, and included all homozygous carriers of the BChE K allele and heterozygous carriers of BChE A and F alleles. The identified interaction of BChE genotype with veterans’ use of PB was a singular observation and requires corroboration in other veteran groups. However, this initial finding comes from a representative sample of Gulf War veterans, was highly significant, and was not explainable by study factors such as differential reporting of PB use or how GWI cases were defined.

This study therefore provides a foundation and model for an expanded investigation of gene-exposure interactions that potentially explain why some military personnel developed chronic illness in connection with Gulf War service while others, with similar deployment experiences and exposures, remained healthy. The association of GWI with BChE genotype was specific to veterans who reported using PB in our sample, and did not occur in connection with other wartime experiences like hearing chemical alarms and using different types of pesticides. Multiple genes can potentially contribute to differences in individual responses to cholinesterase inhibitors and other chemical exposures [41, 42]. It is therefore possible that other genetic factors, such as polymorphisms of the paraoxonase (PON1) enzyme, might have played a role in the development of chronic illness in connection with Gulf War exposures.

Genetic variability in biological processes that neutralize AChE inhibitors has long been hypothesized to underlie differences in vulnerability to these compounds—in Gulf War veterans and in other exposed populations [2, 28, 43, 44]. Although this is the first study to demonstrate a specific gene-exposure interaction in the risk for GWI, our findings are consistent with preliminary indications that unanticipated effects of Gulf War exposures may be linked to BChE genetic variability.

A 1995 case report described the experience of an Israeli soldier who developed severe symptoms when taking PB during the Gulf War at the recommended dosage (30 mg., three times per day) [31]. This soldier was found to be homozygous for the BChE A allele, and had a history of post-anesthesia apnea. Further investigation revealed that his serum BChE had only one-third normal activity, and very poor binding capacity for carbamates, including PB. Later, results from a DOD-sponsored study described in a federal project report suggested that BChE genotype may be associated with chronic symptoms in Nebraska Gulf War veterans [45]. Investigators determined BChE allele frequencies in 226 Gulf War veterans. Of 11 carriers of the A or F alleles, eight (73%) had previously identified themselves as GWI “cases,” suggesting a potential link between GWI and BChE variants.

Our general finding that mean BChE enzyme activity, measured at the time of the study, did not differ between GWI cases and controls parallels results from two previous studies [46, 47]. However, a 2000 study indicated that BChE activity was significantly *higher* in 152 British Gulf War veterans with health concerns, compared to age and sex-matched individuals in the general population [48].

Variability in PON1 has also been suggested as a possible contributing factor to GWI. Four studies have reported on one or more PON1-related variables in relation to Gulf War service, yielding a somewhat mixed picture. The first, studying a Navy Seabees unit, reported that the 12 veterans with the most severe symptomatic illness were significantly more likely to have low PON1 type Q arylesterase activity [46]. Two larger studies of British Gulf War veterans indicated that PON1 paraoxon activity was significantly lower in ill Gulf War veterans, overall, than comparison groups [48, 49]. A more recent study of Iowa Gulf War veterans reported no associations with PON1 activity [47]. None of these studies, however, looked specifically at the question of whether associations between GWI and exposures differed with BChE or PON1genotype or phenotype.

### BChE in other exposed populations and in animal models

The physiological functions of BChE are not well understood. Enzyme activity varies with BChE genotype, as demonstrated here, but is also temporarily reduced by certain drugs and environmental exposures. Although BChE genotype has previously been hypothesized to contribute to differences in susceptibility to adverse effects of cholinergic drugs and pesticides [28], few studies have evaluated BChE-associated risk in exposed populations. A 1996 study reported that “non-usual” BChE variants were significantly overrepresented among Brazilian farmers exposed to carbamate and organophosphate pesticides who exhibited mild signs of poisoning [50]. Related findings, from a more recent assessment of pesticide-exposed farm workers, indicate that of 256 candidate single nucleotide polymorphisms (SNPs) in 30 genes potentially related to pesticide metabolism, the strongest correlates of reduced cholinesterase activity, over four time points, were two SNPs in the BChE gene [51].

Studies in several animal models also suggest a role for BChE in providing a protective biological response to PB [52, 53]. This includes findings that delayed central nervous system effects resulting from low-dose PB exposure occurred only in a genetic strain of rats with reduced BChE activity levels, not in a standard strain with normal BChE activity [54, 55]. Additional insights regarding BChE activity in the brain come from studies suggesting that BChE has physiological actions in addition to its scavenging function. These include indications that BChE becomes biologically active in the brain under conditions in which acetylcholine is elevated, acting directly to reduce acetylcholine levels [56–58].

### Current BChE findings in context

Several aspects of our findings deserve emphasis. First, the association between BChE genotype and GWI was not identified when all Gulf War veterans were evaluated together, but was only apparent in relation to PB use. This underscores the importance of assessing risk factors for GWI, including genetic risk factors, in appropriate subgroups. It also illustrates an important principle that is too often overlooked: genetic polymorphisms that produce reduced capacity to neutralize specific chemicals would generally not be expected to produce health consequences for individuals who are not exposed to those chemicals. It is therefore essential that studies whose purpose is to assess genetic factors in relation to GWI carefully evaluate those associations in connection with relevant exposures.

It is important to note that GWI was also associated with PB use in veterans with the common U/U and U/K genotypes in our sample, although the association was not as strong as in the BChE-LCV subgroup. This indicates that PB was a risk factor for GWI for reasons other than the apparent genetic vulnerability identified in this study. As detailed in a 2008 federal committee report, *Gulf War Illness and the Health of Gulf War Veterans*, PB is the most consistently-identified risk factor for GWI across all epidemiologic studies that adjusted for effects of concurrent wartime exposures [2]. This broader role for PB as a risk factor for GWI is supported by identification of dose–response effects in several studies [25, 26, 59], and by studies linking PB use during the Gulf War to brain and hypothalamic-pituitary-adrenal alterations in Gulf War veterans evaluated many years later [9, 18, 19].

The association of PB with GWI differed by BChE genotype, but was not associated with BChE enzyme activity measured at the time of the study. While genetic type is the most sustained correlate of BChE activity over time, it is just one factor that potentially contributed to reduced BChE activity levels in theater. Independent of genotype, it is likely that BChE activity levels were reduced, to varying degrees, among some Gulf War personnel in theater, particularly during periods of sustained exposure to pesticides, PB, and other cholinesterase inhibitors. Such “situational” reductions in BChE activity might have rendered veterans vulnerable to developing chronic symptoms via mechanisms similar to those that contributed to GWI in the BChE-LCV subgroup of veterans.

Our finding of a significant interaction between PB and BChE genotype in the risk for GWI can only be considered preliminary, since it has not been evaluated in other veteran groups. If corroborated, this interaction would apply to only a subgroup of veterans who developed GWI. It is not possible to accurately predict the number of GWI cases associated with this interaction. A very speculative estimate, based on our results, would rely on our observation that among all veterans in our sample who carried “at risk” BChE genotypes and also used PB during the war, 80% became GWI cases, constituting 9% of all cases in our study. If, overall, 9% of the 175,000-233,000 U.S. Gulf War veterans with GWI [2, 4, 20] became ill as a result of an interaction between PB use and BChE genotype, an upper limit of 16,000-21,000 affected veterans might be inferred. This estimate is tempered by uncertainties surrounding the diversity of exposures encountered by veterans in theater and additional genetic factors that potentially contributed to veterans’ risk of developing GWI.

Our study had several limitations. Most importantly, our sample was only large enough to include 28 veterans with the less common BChE genotypes, disallowing a detailed assessment of the risk associated with individual genotypes, or in-depth multivariable assessment of all exposures in this subgroup. In this relatively small subgroup, the confidence interval for the observed association was wide, although the point estimate was extremely high (OR = 40.0) and statistically significant. It is doubtful that an association of this magnitude would be an artifact of confounding by another exposure, especially since other potentially cholinergic exposures did not significantly interact with BChE genotype in our sample.

Additional limitations, common to other Gulf War studies, include our use of self-reported symptoms and deployment exposures, the only methods currently available to assess GWI and most aspects of Gulf War deployment. Usual concerns related to recall bias as an explanation for exposure-illness associations in Gulf War studies are less a concern for our findings, however, which relied on objective laboratory measures of both BChE genotype and activity. Veterans were unaware of their BChE genotype in reporting both symptoms and exposures, and did not report PB use differentially by genotype.

An important strength of our study is the sample, drawn randomly from a defined population. This provides confidence that our findings are more likely to be comparable to the larger Gulf War veteran population than findings from registry or clinical samples. Our results also indicate that use of the Kansas GWI case definition to determine case status was a strength. The Kansas GWI criteria appear to define a profile of symptomatic illness that is more specific to service in the 1991 Gulf War than other case definitions in use [1, 21].

Overall, our finding of a significant interaction between PB use and BChE genotype in the risk for GWI is provocative, and possibly of great importance. If corroborated, it indicates that susceptible military personnel should not take PB, just as some individuals are currently not given succinylcholine when a genetic deficiency of BChE is suspected. This potentially has far reaching implications for U.S. military policy, which currently directs prophylactic use of PB as a protective measure for troops who are at risk for exposure to the nerve agent soman [60].

## Conclusions

The specific causes and biological nature of Gulf War illness have yet to be adequately characterized. Our results illustrate complexities inherent in identifying either genetic factors or wartime exposures as risk factors for GWI and the need to assess both. Findings from this and related studies indicate that the long-sought “smoking gun” pointing to the cause of GWI may differ for different subgroups of veterans. Certainly, the magnitude of the association of GWI with the gene-exposure interaction identified here, if replicated in other groups, indicates that PB is a strong “smoking gun” candidate for the subgroup of Gulf War veterans with identified BChE genotypes.

## Authors’ information

Mary R Cook is currently retired.

## Abbreviations

AChE: Acetylcholinesterase
BChE: Butyrylcholinesterase
BChE-LCV: Butyrylcholinesterase-less common variants subgroup
CI: Confidence interval
CDC: U.S. Centers for Disease Control and Prevention
DOD: U.S. Department of Defense
GWI: Gulf War illness
OR: Odds ratio
PB: Pyridostigmine bromide
PON1: Paraoxonase
SD: Standard deviation.
